# Social determinants of inadequate prenatal care utilization in sheltered homeless mothers in the Greater Paris area in France

**DOI:** 10.3389/fpubh.2023.1080594

**Published:** 2023-03-21

**Authors:** Elodie Richard, Cécile Vuillermoz, Sandrine Lioret, Raquel Rico Berrocal, Emmanuelle Guyavarch, Yann Lambert, Elie Azria, Karen Leffondre, Stéphanie Vandentorren

**Affiliations:** ^1^Santé Publique France, University of Bordeaux, Bordeaux Population Health Laboratory, INSERM U1219, Bordeaux, France; ^2^Sorbonne Université, INSERM, Institut Pierre Louis d'Epidémiologie et de Santé Publique (IPLESP), Department of Social Epidemiology, Paris, France; ^3^Université de Paris, CRESS, INSERM, INRAE, Paris, France; ^4^École des Hautes Études en Sciences Sociales (EHESS), Center d'étude des Mouvements Sociaux (CEMS), EHESS/CNRS UMR 8044/INSERM U1276, Paris, France; ^5^Observatoire du Samu social de Paris, Paris, France; ^6^CIC Guyane, Cayenne, France; ^7^Maternity Unit, Paris Saint Joseph Hospital, Université de Paris, U1153 CRESS, Obstetrical Perinatal and Pediatric Epidemiology Research Team, EPOPé, INSERM, INRA, Paris, France; ^8^University of Bordeaux, Bordeaux Population Health Laboratory, INSERM U1219, Bordeaux, France

**Keywords:** inadequate prenatal care utilization, homeless, social determinants, inequalities, housing instability

## Abstract

**Background:**

Sheltered homeless families suffer from deleterious living conditions such as housing instability (i.e., moving from one shelter to another) that could be an additional barrier to healthcare utilization. Few studies have specifically examined perinatal health in homeless mothers and their utilization of prenatal healthcare. This study aimed to identify social determinants such as living conditions (i.e., housing instability) associated with inadequate prenatal care utilization (PCU) in sheltered homeless mothers in the Greater Paris area in France.

**Methods:**

The homeless children and families cross-sectional survey [ENFAMS: (Enfants et familles sans logement)] was performed on a random representative sample of homeless families living in shelters in the greater Paris area in 2013. Following French guidelines, PCU was deemed inadequate if one or more of the following criteria was met: attending fewer than 50% of recommended prenatal visits, PCU initiation after the first trimester of pregnancy, and fewer than three ultrasounds during the entire pregnancy. Families were interviewed in 17 languages by trained peer interviewers in face-to-face interviews. Structural equation modeling was used to identify factors associated with inadequate PCU and to estimate correlations between them.

**Results:**

This study analyzed data on 121 homeless sheltered mothers who had at least one child less than one year old. They were socially disadvantaged and most were born outside France. One in five (19.3%) had inadequate PCU. Associated factors were socio-demographic characteristics (young age, primiparous), health status (dissatisfaction with self-perceived general health), and living conditions (housing instability in the second and third trimesters).

**Conclusion:**

It is essential to reduce housing instability to help sheltered mothers to benefit from social, territorial and medical support and healthcare utilization. Housing stability for pregnant sheltered homeless mothers should be a priority to ensure better PCU and guarantee the newborn's health as much as possible.

## Introduction

The profile of homeless people in Europe has been changing for several decades, with an increase in the numbers and proportions of migrants and families with young children (1). According to a homeless survey conducted in 2012, 140,000 people were homeless in metropolitan France (2). Of these, two-thirds were adults, and half lived in the Greater Paris area, representing a 50% increase since 2001 (2). Between 1995 and 2018, the proportion of families with children among the homeless in France increased from 8% to 25% (2–4). For the past 20 years, health services in France have focused principally on providing support for the male homeless population (2). Meeting the specific needs of the female homeless population requires tailor-made services, particularly for pregnant women during the prenatal period. According to the Solidarity Paris Mothers (SOLIPAM) network, the number of requests for accommodation for homeless pregnant women has increased every year since 2010, and almost tripled between 2011 and 2018, from 488 to 1373 (4).

Homeless women have poor obstetrical health indicators (5) and suffer from an accumulation of various social insecurities during the perinatal period that exposes them to extreme vulnerability, such as food insecurity or housing instabilities (frequent move from one shelter to another) that implies physical and psychological issues. Homeless mothers face a high level of perinatal mortality (6, 7) and morbidity, but also inadequate prenatal care utilization (PCU) (5, 6, 8). Homeless mother needs adequate prenatal utilization to prevent perinatal burdens, as most of them are concerned by low birth weight or preterm birth (5, 7, 9). According to a 2015 US study, pregnant homeless women with housing instability were 1.43 times more likely to deliver a low birth weight child than settled women, and 1.24 times more likely to deliver a preterm child (9). Pregnant homeless women with housing instability also have a higher neonatal mortality rate than the general population (5, 9, 10). In France, only one study has investigated living conditions in homeless sheltered families relating to their health's status. This study highlights their housing instability with more frequent moves over the first year of homelessness (11).

Homeless women also face barriers to their utilization of healthcare, including violence, discrimination (12), and situations where this utilization competes with their primary needs (9). They also have difficulty accessing contraception and gynecological care (12, 13). They are less likely to be screened for gynecological cancers and sexually transmitted infections (12, 13) and face barriers to negotiating condom use with their partners (13). They are twice as likely to have an undesired pregnancy as settled women (8, 14).

Prenatal care anticipates, prevents, and ensures mother and child health (15). It limits obstetric complications during delivery (15, 16). Disparities in PCU can be seen in high-income countries, for example, between native-born and immigrant women (17, 18) and between women living in poverty and those who are not (19). Social factors could explain it, such as young age, low education level, country of origin, undesired pregnancy (12, 20–22), low socioeconomic status (12, 19, 23), as well as financial, organizational and transportation difficulties in accessing care (12, 20).

Studies investigating social determinants associated with inadequate PCU in socially disadvantaged populations have mainly focused on the factors mentioned above. However, some factors remain understudied, such as housing instability, which is a known stressor in this population in high-income countries (1, 2, 11, 24). The present study aimed to identify social factors associated with inadequate PCU in sheltered homeless mothers in the Greater Paris area in France, particularly deleterious living conditions, such as housing instability.

## Methods

### Survey design and study population

The homeless children and families cross-sectional survey (ENFAMS) was the first epidemiological study in France of sheltered homeless families (11). This survey was conducted in 2013, included a representative sample of families living in the Greater Paris area and was implemented by the “Observatoire du SAMUSOCIAL.” Eligibility criteria were being a parent aged >18 years, who had at least one child under 13 years, was sheltered in the Paris area in a social hotel, emergency center, asylum seekers' center, or long-term rehabilitation center, was able to speak one of the 17 languages covered in the study, and provided informed consent to participate. The sampling design included three levels of random sampling: shelters, families, and one child from every family (11). The present study included all sheltered homeless women in ENFAMS with a child under 1 year of age who had started prenatal care in France.

### Data collection

A standardized face-to-face questionnaire was administered to parents by a bilingual interviewer (French and the respondent's language).

The questionnaire collected demographic and socioeconomic data, as well as data on housing history since the first episode of homelessness, migration trajectory, social relationships and social support, food insecurity [assessed using the Household Food Security Survey (HFSSM) (25)], health perceptions and self-reported health using the Health Perceptions Questionnaire (26) from the Health, inequality and social disruption cohort study (SIRS : Santé, inégalités et ruptures sociales) (27), mental health [assessed using the World Health Organization's CIDI-S (28) to measure depressive symptoms and the MINI-S (29) to assess post-traumatic stress disorder (PTSD)], and lastly, healthcare utilization (type and locations of health services visited). The perinatal care indicators studied were those from the 2010 National Perinatal Survey (30).

To assess food insecurity and PTSD, we used standardized questionnaires in the following way:

- The HFSSM questionnaire includes ten questions. Two scores equaling the total number of affirmative responses to the questions were calculated at household level. The household score was divided into four categories defined by the usual thresholds: food security (score: 0–2), food insecurity without hunger (3–7), food insecurity with moderate hunger (8–12) and food insecurity with severe hunger (13–18, 31).
- The MINI-S questionnaires include nine questions. If the participant answers “no” to the first two questions, the scale does not show PTSD. If “yes” to the first two questions AND a total of three “yes” to the following questions, or “yes” to one of the first two questions AND a total of four “yes” for the following questions, then the scale detects PTSD (29).

### Prenatal care utilization

PCU was defined based on the French National Authority for Health's (HAS) recommendations (32), explicitly initiating prenatal care in the first trimester of pregnancy, followed by monthly prenatal consultation and three ultrasounds (in each trimester of pregnancy) (32). PCU was deemed inadequate if one or more of the following was true: fewer than 50% of recommended prenatal visits (i.e., for a full-term pregnancy, inadequate PCU included fewer than three prenatal visits or, for a 7-month premature pregnancy, fewer than two consultations), PCU initiation after the first trimester of pregnancy, and fewer than three ultrasounds during the entire pregnancy (32).

### Factors associated with inadequate PCU

**Figure 1:**
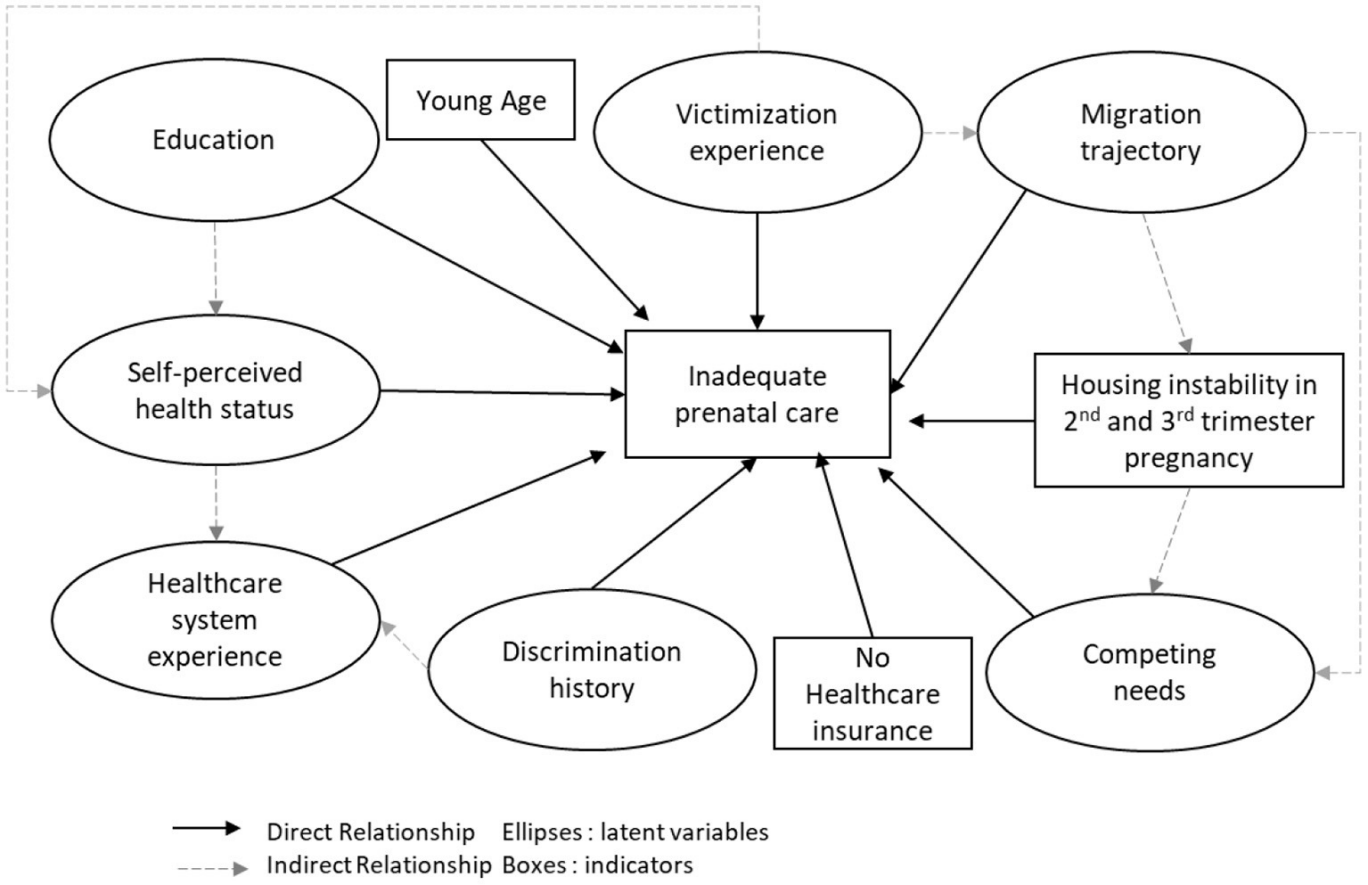
Conceptual model of inadequate PCU of homeless mothers. ENFAMS cross-sectional survey, Greater Paris area, 2013.

The conceptual model, illustrated in Figure 1, highlights the relationships identified a priori. In order to test the relevance of applying this conceptual model to our study, we used Structural Equation Modeling (SEM) to analyse complex relationships between variables of several domains (33). SEM simultaneously estimates several regression models reflecting all the hypotheses of a conceptual model represented on a graph where observed and unobserved latent variables are distinguished. A latent variable is an unobserved variable representing a concept expressed through a set of observed variables called indicators. Latent variables represent the covariance of these indicators.

#### Conceptual model

To develop the conceptual model, we have proceeded in two steps. First, we used “Behavior Model For Health Service Use” (34) adapted by Vuillermoz et al. conceptual model to consider the determinants of unmet healthcare needs in homeless people in the ENFAMS survey (35). The “Behavioral Model for Health Service Use” conceptual model was developed in 1968 by Andersen et al. (34). It defines factors which predispose a person to use health services (“predisposing factors”), factors favoring (or limiting) their utilization (“enabling factors”) and the individual's care needs (“need factors”) (34). Second, we have included the determinants of prenatal care in socially disadvantaged mother identified in the literature.

Indeed previous studies in socially-disadvantaged women identified several factors associated with inadequate PCU, including young age (8), a low level of education (17, 18), and not understanding, reading or speaking the host country language (36). Migration trajectory has also been reported as a factor for inadequate PCU (18, 20), specifically regarding birthplace and recent immigration (20). Furthermore, a lack of financial resources combined with the absence of health insurance (37), spatial difficulties accessing care (38), and competing primary needs (39) which limit the time available to take an interest in one's pregnancy, and therefore in PCU, have also been identified.

Healthcare needs depend on medical and psychological history such as PTSD (40) or experiencing violence. Other factors not specific to PCU but to primary healthcare utilization should also be considered, such as underestimated self-perceived health status (41), fear of discrimination (35), and lack of experience with the healthcare system.

All these factors represented our initial hypotheses for the model to be tested, except spatial difficulties of access to care, as we considered housing instability during pregnancy a more relevant geographical accessibility factor for homeless women.

#### Construction of the conceptual model

We identified 17 indicators (observed variables from the questionnaire), seven latent variables and three observed variables that did not indicate a latent variable (Figure 1).

We included three observed variables that did not indicate a latent variable in the model: housing instability during the second and third trimesters of pregnancy (i.e., moving home at least once between the fourth month of pregnancy and childbirth) (Yes/No), age at the beginning of pregnancy (continuous variable), and general healthcare insurance (i.e., standard not complementary health coverage) (Yes/No). Table 1 explains how the latent variable has been constructed with the 17 indicators.

**Table 1:** Latent variables construction.

**Latent variables**	**No**	**Indicators**	
1. Education	1	Level of education	Higher/secondary/primary/none
	2	Understanding, reading or speaking the French language	Yes/no
2. Migration trajectory	3	African origin^*^	Yes/no
	4	Time in France before the beginning of the pregnancy	Time in France >6 months vs. <6 months
3. Competing needs	5	Number of children under 3 years of age	1–2 children vs. >2 children
	6	Food insecurity	Mild/moderate vs. severe
	7	Single mother	Yes/no
4. History of victimization	8	Symptoms of PTSD over the previous 12 months	Yes/no
	9	Lifetime sexual abuse	Yes/no
5. Discrimination history	10	Refusal of free state-based medical aid (AME) and free complementary health insurance for low-income earners (CMU)	Yes/no
	11	Unsatisfactory care provided by a health professional^**^	Yes/no
6. Healthcare system experience	12	History of gynecological visits	Yes/no
	13	Parity	0/at least one delivery
	14	Unmet healthcare needs^***^	Yes/no
7. Self-perceived health status	15	Self-perceived psychological	Very poor/poor vs. moderate vs. good/very good
	16	Self-perceived physical health state	
	17	Self-perceived general health state	

* Yes: African origin, No: Europe, south America, Asia and French origin.

** “Have you ever been treated worse by a doctor or medical staff than other patients?” Care was unsatisfactory if the participant responded “yes”.

*** i.e., Having foregone at least one planned healthcare intervention in the previous 12 months.

### Statistical analysis

A descriptive analysis of all participant characteristics was performed. All proportions were inversely weighted to each participant's inclusion probability following the sampling design (11). We compared the sample with complete data against the sample containing missing data.

To study factors associated with inadequate PCU, we used SEM. First, we analyzed each latent variable's weighted covariance matrix of indicators with the Pearson correlation coefficient. All variables with a coefficient lower than 0.30 were excluded (42).

Second, we ensured the unidimensionality of each latent variable using a scree plot (42). Third, we performed confirmatory factor analysis (CFA) to estimate relationships between indicators from each latent variable (42). More specifically, CFA allowed us to evaluate the share of variance explained by each indicator within their latent variable. We also checked the goodness of fit of the model. Depending on their level of theoretical relevance, non-significant indicators at 5% were either excluded from the latent variable or reintegrated into the model as observed variables not indicative of a latent variable.

Finally, we studied the structural model comprising latent variables and observed variables that were not indicative of latent variables. We checked for model identifiability. For an SEM model to be identified, the number of observations must be greater than the number of parameters to be estimated. The latter corresponds to the number of elements constituting the variance/covariance matrix of the indicators, or *p* (*p* + 1) /2, where p is the number of indicators. Our final model identified 11 indicators, or a minimum of 66 (11^*^12/2) observations required. As we had 121 observations, we were able to test the model. This was done using the Weighted Least Squares with Mean and Variance adjustment (WLSMV) estimator, which is used when models include binary or ordinal variables (43, 44). All factor loadings were weighted according to the inverse of the inclusion probability of each participant. The significance level was set at 5%.

We checked the goodness of fit of the model using the comparative fit index (CFI > 0.90) and the mean quadratic approximation error (RMSEA < 0.08) (33). All analyses were conducted using R software version 3.2.1 with the “lavaan.survey” function.

## Results

### Description of the study population

**Figure 2:**
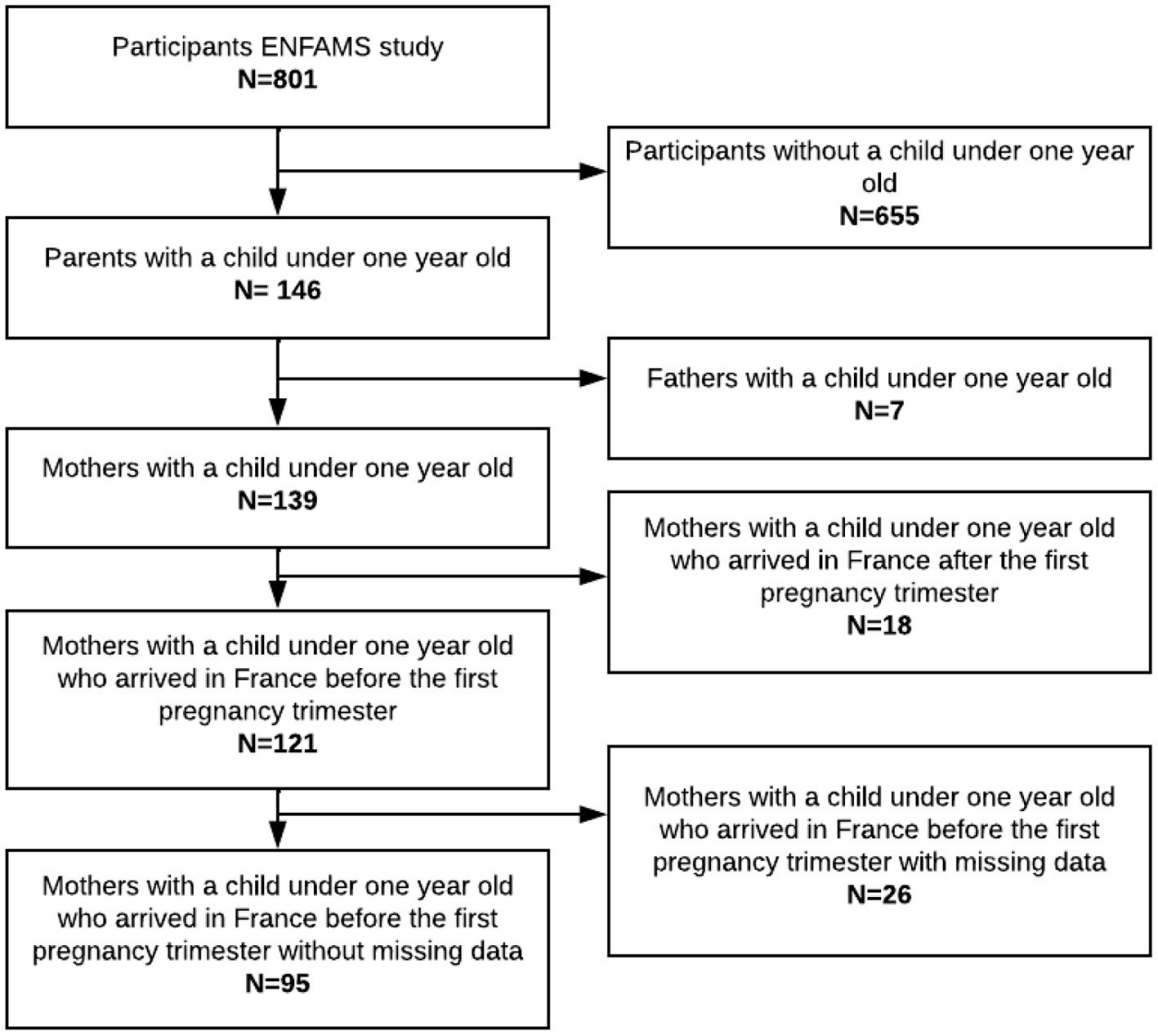
Flowchart of PCU ENFAMS cross-sectional study, Greater Paris area, 2013.

ENFAMS study was performed from January to May 2013. Among the 801 participants from homeless families included in ENFAMS, 121 mothers met the present study's eligibility criteria, of whom 95 had complete data (Figure 2). All included participants have missing data on one or two random variables.

### Participant characteristics

Participants' characteristics are presented in Table 2. Participants were 29.7 years old on average. One-third were single. Three-quarters had at least secondary level education, and half had difficulty understanding, reading, or speaking French. The mean monthly household income was 282.40 €/Consummation Unit.^1^ Nine in ten women were born outside France, and 8 % had immigrated less than 6 months before the beginning of their pregnancy. Half declared moderate or severe food insecurity. One in five presented PTSD in the previous 12 months, and 14 % had been sexually abused. Half had been homeless before their pregnancy, 39% during pregnancy, and 10% after delivery. More than two-thirds had housing instability in 2nd and 3rd trimesters of pregnancy.

**Table 2:** Characteristics of sheltered homeless mothers with at least one child under 1 year old (*N* = 121). ENFAMS cross-sectional survey, Greater Paris area, 2013.

**Characteristics**	**Participants (*****N*** = **121)**		
	***N***	**%** ^*^	**95% CI** ^***^
		**Mean** ^**^	
Age at the beginning of pregnancy (in years)^**^	121	29.7	(27.5, 31.9)
**Education level**			
- Never attended school	16	12.0	(3.9, 20.0)
- Primary	10	8.7	(3.2, 14.2)
- Secondary	61	56.6	(38.7, 74.2)
- Higher	21	22.7	(3.9, 41.4)
Difficult to understand, read, or speak French	59	55.7	(41.0, 69.7)
**Professional status**			
- Employed	7	13.4	(0.0, 28.3)
- Unemployed	34	30.8	(18.8, 42.8)
- Inactive	71	55.8	(40.0, 71.6)
Monthly income per consumption unit (CU) (in euros/CU)^**^	121	282.4	(189.7, 375.1)
**Health insurance**			
- No	12	10.0	(3.6, 16.4)
- AME^a^	40	33.4	(19.6, 47.3)
- CMU^a^	55	48.2	(30.5, 65.9)
- General social security	5	8.3	(2.6, 14.1)
**Time of the first episode of homelessness**			
- More than a year before pregnancy	24	34.0	(15.4, 52.6)
- Less than a year before pregnancy	24	16.9	(8.3, 25.4)
- In the 1st trimester of pregnancy	22	14.8	(4.6, 24.9)
- In the 2nd trimester of pregnancy	16	8.5	(3.2, 13.9)
- In the 3rd trimester of pregnancy	17	15.4	(7.2, 23.6)
- After delivery	9	10.4	(4.2, 16.6)
Housing instability in the 2nd and 3rd trimesters of pregnancy^****^	94	71.0	(54.5, 88.6)
**Mother's birthplace**			
- France	15	8.5	(3.9, 13.1)
- Outside of France	106	91.5	(86.9, 96.1)
**Administrative status**			
- Not regularized	40	35.3	(21.7, 48.9)
- Regularized	57	53.2	(38.0, 68.4)
- French citizen	15	11.5	(6.1, 16.8)
**Length of immigration before the beginning of pregnancy**			
- <6 months	43	8.5	(3.9, 13.5)
- >6 months	57	68.8	(56.7, 80.8)
- French citizen	15	22.7	(12.5, 32.9)
Two or more children under 3 years of age living with mother	19	26.4	(13.8, 39.1)
**Food insecurity**			
- Low	52	51.8	(34.5, 69.1)
- Moderate or severe	53	48.2	(30.9, 65.5)
Single mother	52	33.4	(19.8, 47.1)
PTSD in the previous 12 months	22	18.8	(7.4, 30.2)
Sexually abused	20	14.3	(4.6, 24.0)
Refused for CMU^a^ and/or AME^a^	29	11.7	(4.2, 19.2)
Unsatisfactory care by a health practitioner	18	25.3	(13.2, 37.4)
**Self-perceived general health status**			
- Very poor/poor	7	6.6	(0.0, 15.4)
- Moderate	38	37.0	(19.8, 54.1)
- Good/very good	67	56.4	(41.5, 68.2)
Primiparous	47	31.0	(19.5, 42.4)
No gynecological visit	35	27.6	(15.0, 40.3)
Unmet healthcare needs	25	20.8	(10.9, 30.8)
Spatial difficulties in accessing care	56	43.3	(10.9, 50.8)

* Weighted values for qualitative variable.

** Weighted values for quantitative variable.

*** 95% Confidence interval.

**** Moved home at least once.

a AME, free state-based medical aids; CMU, free complementary health insurance for low-income earners.

The characteristics of the present study's participants did not differ from those of ENFAMS participants with missing data (details given in Supplementary Table S1).

### Inadequate PCU

The inadequate PCU in our ENFAMS study sample was 19.3% (Table 3). Just over three percent (3.3%) had no PCU, while 13.5% had initiated PCU after the first trimester. Concerning the delivery term, 11.1% of participants had fewer visits than recommended. Finally, 9.6 % had fewer than three ultrasounds during their pregnancy.

**Table 3:** Description of sheltered homeless mothers' PCU (*N* = 121), ENFAMS cross-sectional survey, Greater Paris area, 2013.

**Inadequate PCU**	**Participants (*****N*** = **121)**		
	***N***	**%** ^*^	**95% CI** ^**^
Inadequate PCU^***^	30	19.3	(9.4, 29.1)
No PCU	4	3.3	(0.2, 6.5)
PCU initiation after the 1st trimester	22	13.5	(6.5, 20.5)
Fewer visits than recommended relative to their pregnancy term	19	11.1	(4.5, 17.7)
Fewer than three ultrasounds during their pregnancy	13	9.6	(2.6, 16.6)

* Weighted values.

** 95% confidence interval.

** If one or more of the following was true: fewer than 50% of recommended prenatal visits, PCU initiation after the first trimester of pregnancy, and fewer than three ultrasounds during the entire pregnancy.

### Validation of latent variables

The weighted correlations between the observed variables of each latent variable ranged from 0.01 to 0.52 (details given in Supplementary Table S2). The following elements were not retained for the analysis: the “education” and “history of discrimination” latent variables, the “having children under three years old” indicator from the “competing needs” latent variable, and the indicator “unsatisfied physical self-perceived health status” from the latent variable “self-perceived health status.” All scree plots checking the unidimensionality of each latent variable were satisfactory: all curves leveled off above 1, which indicated that each latent construct was indeed unidimensional (details given in Supplementary Figure S1).

### Measurement models

CFA estimated four latent variables. Of these, “victimization experience,” “migration trajectory,” and “competing needs” had factor loadings between the latent variable in question and its indicators which were statistically significant at the 0.05 level. The fourth, “self-perceived health status” did not, and was therefore excluded.

### Structural model

**Figure 3:**
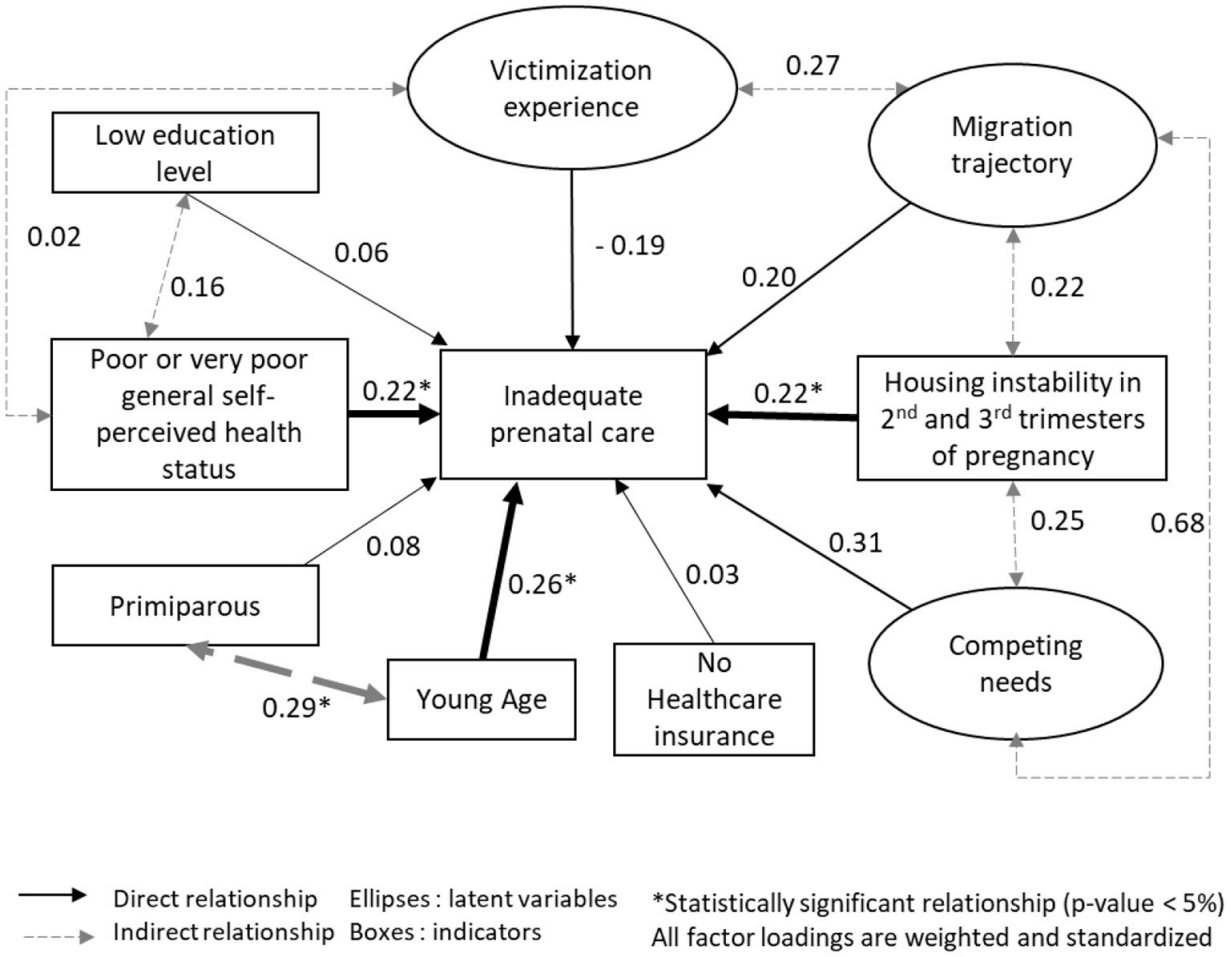
Final SEM model of inadequate PCU in sheltered homeless mothers (N=95), ENFAMS cross-sectional survey, Greater Paris area, 2013.

General self-perceived health status, education level, and being primiparous were included in the final SEM because of their theoretical relevance as variables not indicative of latent variables. The final SEM model (Figure 3) identified that housing instability in the 2nd and 3rd trimesters [β = 0.22, 95% CI (0.06, 0.39)], young age [β = 0.26, 95% CI (0.01, 0.54)], and poor and very poor general self-perceived health status [β = 0.22, 95% CI (0.01, 0.44)], were positively and significantly correlated with inadequate PCU. Young age was positively and significantly correlated with being primiparous [β = 0.29, 95% CI (0.02, 0.57)]. No other relationship was statistically significant. The goodness-of-fit indices of the final model were acceptable, with a CFI of 0.63 and an RSMEA of 0.08 [95% CI (0.06, 0.12)].

## Discussion

In our study, inadequate PCU in sheltered homeless mothers in the Greater Paris area was higher (19.3%) than in the general population of mothers in various high-income countries. A study conducted by the Center for Disease Control and Prevention in the United States between 1995 and 2002 estimated inadequate PCU (using the Adequacy of Prenatal Care Utilization (APNCU) index^2^) at 11.2% among a study population of 30 million pregnant women (23). Another study conducted in the Netherlands in 2008 found that 14.8% of the general population of pregnant women had inadequate PCU (APNCU index) (38).

However, our result was lower than that found in several other studies. For example, in the Netherlands and Spain, inadequate PCU was estimated at 23% in immigrant women (APNCU index for both countries) (17, 18). The 2017 French prospective cohort study PréCARE, which targeted women living in socioeconomically disadvantaged situations, found values of 26.9% and 34.7% (based on the HAS definition) in participants born in North and Sub-Saharan Africa, respectively (20). Another French study conducted in 2013 estimated inadequate PCU (HAS) in 25% of women living in the Ile-de-France region (which includes Greater Paris) and Seine-Saint-Denis (an area inside Greater Paris) (45).

In the 2013 ENFAMS survey, inadequate PCU was slightly higher than in the 2016 National Perinatal Survey (NPS) (19.3% vs. 17.8%). In the latter, 18.2% of all mothers had fewer visits than recommended during their pregnancy (vs. 11% in ENFAMS), 7.6% initiated care after the first trimester (vs. 13.5%), and 1% had fewer than three ultrasounds during their pregnancy (vs. 9.6%) (46). These results suggest that the primary problems of PCU utilization by sheltered homeless mothers are care initiation (i.e., first contact with a health professional) and subsequent care for complementary examinations. An ethnographic survey of mothers who participated in ENFAMS (47) highlighted several difficulties regarding PCU initiation linked to a lack of knowledge about where to go for follow-up care, including ultrasounds. This could partly explain this population's fewer ultrasounds and late PCU initiation. Another possible reason for these problems is that sheltered homeless mothers are often discriminated against and do not feel welcome in healthcare reception services. Although many of the sheltered homeless mothers in the ENFAMS study had changed accommodation several times to areas outside the Ile-de-France region, most of their visits occurred in five maternity hospitals inside the region (47).

Our results highlight that being a young mother, being primiparous, having a poor or very poor general self-perceived health status, and having housing instability in the second and third trimesters of pregnancy, were the primary factors for inadequate PCU. The finding for young mothers reflects the literature (8). As expected, inadequate PCU was even higher in primiparous young mothers. Self-perceived health status refers to one of the dimensions of healthcare utilization. Healthcare utilization integrates the need for care, the intention to consult, and using the healthcare system effectively (34). Healthcare utilization refers to people's perceptions of the need for care and their ability to obtain appropriate care services without delays or obstacles. In the ENFAMS survey, the more mothers were unsatisfied with their self-perceived health status, the more they were likely to have inadequate PCU. This highlights the difficulties of sheltered homeless mothers in overcoming barriers to achieving PCU.

The significant barrier was housing instability in the 2nd and 3rd trimester of pregnancy. Housing stability allows sheltered homeless mothers to develop territorial and social networking and support to satisfy their primary survival needs (47). Housing instability is a broader concept than homelessness (48, 49). It captures the notion of territorial networking, which is essential for access to care (knowledge of care structures, obtaining regular appointments, etc.). This territorial and social networking improves mothers in two ways (47–49). First, it helps them and their families create and maintain a supportive social environment and links it to other community services. This facilitates regular parenting support and practical help for mothers under stress and facing major barriers (48, 49). Second, territorial networking encourages self-confidence, empowerment, and the mother-to-be's physical, emotional, and psychological availability to participate in PCU. This helps strengthen the mother-child bond (47). Sheltered homeless mothers facing housing instability in the 2nd and 3rd trimesters may not have enough opportunities, before delivery, to develop a territorial and social network to satisfy all primary survival needs (44).

Sheltered homeless mothers should be provided special attention given the difficulties they face in overcoming barriers to PCU. Specifically, advocating housing stability during pregnancy is crucial, as results from other studies and data from community associations (7, 8, 14, 49, 50) highlight that housing stability helps limit medical wandering (48) and promotes “territorial networking” of pregnant women in their current living environment (47). This improves PCU, limits the risk of obstetrical complications, and fosters maternal-fetal health while limiting inequalities in PCU (19, 49).

This study has limitations. First, this study tried to maintain a temporal sequence to ensure that the dependent variables influenced inadequate PCU, not the reverse. We constructed the variable “housing instability in the second and third trimesters” of pregnancy. This variable was concomitant to PCU to capture the concept of territorial networking during pregnancy. Consequently, participants with housing instability in the second and third trimesters may have been overrepresented. Given the extremely socially disadvantaged situation in which homeless mothers live, we assume that any misclassification was non-differential between the two types of PCU (i.e., inadequate and optimal). Therefore, we may have limited the evidence of some associations. Second, we used the French recommendations to construct the indicator of inadequate PCU. This allows us to compare our results with the French studies, especially the PreCare cohort (19). It also allows us to study the initiation of inadequate prenatal care and the utilization of complementary prenatal examinations. However, the French recommendations require more than the international ones, such as the world health organization. Not all women should be aware of the eight consultations in France instead of four internationally recommended and three ultrasound exams during pregnancy. Inadequate PCU was a quantitative indicator of care consumption in this study. The quality of care, despite its importance in this context, was not considered. Third, we did not measure participants' cultural adjustment to PCU in France, while most are foreigners and have lived in countries that do not apply the same recommendations in terms of PCU. Indeed, mothers in very socially disadvantaged situations need a good understanding of current PCU recommendations and the healthcare system to adjust themselves culturally regarding survival needs. Otherwise, they could stop or delay healthcare utilization, especially for immigrants who must also overcome cultural and identity barriers (46). Finally, restricting study selection to mothers of children under 1 year old limited the sample size, which resulted in a significant loss of power in the statistical analysis and may have limited the evidence of some associations.

The strengths of this study lie in its unique data. First, using a robust survey design, the ENFAMS survey targeted a seldom-studied population. It provided an overview of the health status of homeless families with children living in the Great Paris area in 2013. Second, the survey methodology generated a high participation rate and good representativeness of the homeless population. Third, the large panel of comprehensive data collected improved our understanding of the characteristics of the study population. Moreover, this first exploration of the social determinants of PCU by sheltered homeless pregnant women in France was conducted in 17 languages. Finally, we have used an integrative conceptual model adapted to the unmet needs care and prenatal care determinants. Our results fit the conceptual model and were similar to previous studies: we identify age and primiparity as determinants of inadequate PCU. We also identify that poor perceived health status is a determinant of PCU. Housing instability was the significant structural barrier found here, as in other studies on healthcare utilization among socially disadvantaged populations.

Although ENFAMS was conducted in 2013, the situation of homeless families in France has not changed and is even getting worse. According to field-based reports, the number of homeless families in 2021 in the greater Paris area was approximately 1,000 each night, as opposed to 100 in 2013. Moreover, the waiting time between the first request for accommodation and access significantly increased between 2013 and 2021 and can often be very long. The study population here is sheltered homeless women in hotels or emergency shelters. These accommodations are granted for a given time, from one night to a few weeks. For long-term accommodation, people need to have access to social housing. We recommended that as soon as a sheltered homeless woman declares her pregnancy, she should be **sheltered in the same shelter throughout her pregnancy** (R1). Moreover, adequate accommodation for the mother and child (i.e., with kitchen facilities, close to essential services, ...) should be arranged in social housing with **personalized support** like **supportive collaborative practices by social and health workers as soon as possible during pregnancy** (R2). This can be compared to the housing first intervention in the field of mental health.

To improve personalized support for sheltered homeless mothers (R2), we need to **implement early intervention adapted to their needs in prenatal care or promote better conditions for their realization** (R3). For example, we detail some early interventions in prenatal care below. The early prenatal visit (EPP) during the fourth month of pregnancy objectifies the mother and the couple's family, socioeconomic and mental health problems to organize more personalized support (R3.1). However, efforts should continue to ensure that it is achieved for all pregnant women [according to the 2016 NPS, only 28.5 % of mothers had an EPP in 2016 (46)] and promote the conditions for its realization. These conditions could be (i) informing all pregnant women at the beginning of their prenatal care utilization and in migrant social network, (ii) maintaining financial access to EPP and access its realization with human resources (midwives) necessary to carry out in hospitals, (iii) organize the procedures for alerting and bringing social and health worker together (psychosocial medical staff, multidisciplinary consultation meeting, perinatal network) to provide a solution before birth, (iv) valuing collaborative practices through financial incentives or time to ensure social and health worker's participation. Furthermore, social and health workers in the perinatal field must benefit from training on the characteristics of socially disadvantaged populations to better understand this population and identify situations at risk of an interruption in care throughout the pregnancy (R3.2). Personalized support must be reinforced and focused on implementing by developing multi-professional supportive collaborative practices like the SOLIPAM network, regional perinatal network, or psychosocial medical staff (R3.3). A one-stop social and health center, close to where homeless mothers live, maybe one way to overcome the obstacles to PCU that they encounter (R3.4). All these interventions must be perennially supported, politically and financially, by health institutions in order to be effective.

## Conclusion

The results of the present study underline the importance of promoting housing stability for homeless pregnant mothers, especially during PCU. It is essential to develop territorial networking to help this population build a social, professional or medical network, acquire knowledge to maintain good health status, and have the capacity, motivation, and opportunity to regain power over their lives. Housing stability for pregnant homeless mothers should be a priority to ensure better PCU and guarantee the newborn's health as much as possible.

## Data availability statement

The data analyzed in this study is subject to the following licenses/restrictions: the data set generated/used/analyzed during the current study are available from the corresponding author on reasonable request. Requests to access these datasets should be directed to ER, elodie.richard.2@u-bordeaux.fr.

## Ethics statement

The studies involving human participants were reviewed and approved by the Advisory Committee on Information Processing in Health Research (CCTIRS-agreement ref. 2012 02 06—September 2012), the French National Commission for Commission nationale de l'informatique et des libertés (CNIL-agreement decision DR-2013-147—March 2013) and the Ile-de-France Committee for the protection of individuals (CPP-agreement ref. 12.471—August 2012). The patients/participants provided their written informed consent to participate in this study.

## Author contributions

SV conceived the study. YL did analysis during the earliest stages of the study. YL, ER, SV, and EA worked on the methodology of the inadequate prenatal indicator. ER did all statistical analyses and drafted the first version of manuscript. KL and CV verified the statistical analyses. RB, EG, EA, SL, CV, ER, and SV contributed to the interpretation of data. All authors reviewed the manuscript and approved the final manuscript.

## Footnotes

^1^According to the French National Institute for Statistics and Economic Studies (INSEE), to compare the living standards of households of different size or composition, income is divided by the number of consumption units (CU). These are usually calculated as follows:

- 1 CU for the first adult in the household,
- 0.5 CU for the other persons aged s 14 years of age or older,
- 0.3 CU for children under 14 years of age.

^2^The APNCU index is based on the ratio of observed to expected number of prenatal visits. The expected number of visits is based on the American College of Obstetricians and Gynecologists (ACOG) recommendations. The index also considers the month of initiation of PCU.

## References

[1] Busch-Geertsema V Edgar W O'Sullivan E Pleace N. Homelessness and homeless policies in europe: lessons from research. In: Conference on homelessness. (2010), p. 96.

[2] Mordier B. Introduction de cadrage. Les sans-domicile en France : caractéristiques et principales évolutions entre 2001 et 2012. Econ Stat. (2016) 488:25–35. 10.3406/estat.2016.10709

[3] Brousse C. L'enquête sans-domicile 2001. Paris: Insee Méthodes (2006). p. 13.

[4] SOLIPAM. Rapport d'activité du réseau. Paris: SOLIPAM (2018). p. 78.

[5] Beal AC Redlener I. Enhancing perinatal outcome in homeless women: the challenge of providing comprehensive health care. Semin Perinatol. (1995) 19:307–13. 10.1016/S0146-0005(05)80046-1 8560297

[6] Clark RE Weinreb L Flahive JM Seifert RW. Homelessness contributes to pregnancy complications. Health Aff Proj Hope. (2019) 38:139–46. 10.1377/hlthaff.2018.05156 30615521

[7] Barnu PH. Parcours de soins périnatals et grande précarité: expérience du réseau solipam (Solidarité Paris Maman Île-de-France). Contraste. (2017) 46:189–206. 10.3917/cont.046.0189

[8] Smith D Roberts R. Young parents: the role of housing in understanding social inequality. J Fam Health Care. (2011) 21:20–2. 21485898

[9] Cutts DB Coleman S Black MM Chilton MM Cook JT de Cuba SE. Homelessness during pregnancy: a unique, time-dependent risk factor of birth outcomes. Matern Child Health J. (2015) 19:1276–83. 10.1007/s10995-014-1633-6 25404405

[10] Cheung AM Hwang SW. Risk of death among homeless women: a cohort study and review of the literature. CMAJ Can Med Assoc J. (2004) 170:1243–7. 10.1503/cmaj.1031167 15078846 PMC385354

[11] Vandentorren S Le Méner E Oppenchaim N Arnaud A Jangal C Caum C. Characteristics and health of homeless families: the ENFAMS survey in the Paris region, France 2013. Eur J Public Health. (2016) 26:71–6. 10.1093/eurpub/ckv187 26511600

[12] Gadson A Akpovi E Mehta PK. Exploring the social determinants of racial/ethnic disparities in prenatal care utilization and maternal outcome. Semin Perinatol. (2017) 41:308–17. 10.1053/j.semperi.2017.04.008 28625554

[13] MacKellar DA Valleroy LA Hoffmann JP Glebatis D Lalota M McFarland W. Gender differences in sexual behaviors and factors associated with nonuse of condoms among homeless and runaway youths. AIDS Educ Prev Off Publ Int Soc AIDS Educ. (2000) 12:477–91. 11220501

[14] Shuler PA Gelberg L Davis JE. Characteristics associated with the risk of unintended pregnancy among urban homeless women: use of the Shuler Nurse Practitioner Practice Model in research. J Am Acad Nurse Pract. (1995) 7:13–22. 10.1111/j.1745-7599.1995.tb00988.x 7742062

[15] Organisation Mondiale de la Santé (OMS). Recommandations de l'OMS concernant les soins prénatals pour que la grossesse soit une expérience positive. Genève: OMS (2016). p. 10.

[16] OMS. Rapport décennal du Compte à rebours 2015, Survie de la mère, du nouveau-né et de l'enfant. Genève: OMS (2015). p. 2.

[17] Santibáñez M Paz-Zulueta M Ruiz M Castro I Llorca J. Factors associated with lack of adherence to antenatal care in African immigrant women and Spanish women in northern Spain: the role of social risk factors in combination with language proficiency. Midwifery. (2015) 31:61–7. 10.1016/j.midw.2014.05.012 24972927

[18] Boerleider AW Manniën J van Stenus CMV Wiegers TA Feijen-de Jong EI Spelten ER. Explanatory factors for first and second-generation non-western women's inadequate prenatal care utilisation: a prospective cohort study. BMC Preg Childbirth. (2015) 15:98. 10.1186/s12884-015-0528-x 25895975 PMC4409999

[19] Gonthier C Estellat C Deneux-Tharaux C Blondel B Alfaiate T Schmitz T. Association between maternal social deprivation and prenatal care utilization: the PreCARE cohort study. BMC Preg Childbirth. (2017) 17:126. 10.1186/s12884-017-1310-z 28506217 PMC5433136

[20] Sauvegrain P Stewart Z Gonthier C Saurel-Cubizolles MJ Saucedo M Deneux-Tharaux C. Accés aux soins prénatals et santé maternelle des femmes immigrées. Bull Epidémiol Hebd. (2017) 19-20:389–95.

[21] Heaman M Bayrampour H Kingston D Blondel B Gissler M Roth C. Migrant women's utilization of prenatal care: a systematic review. Matern Child Health J. (2013) 17:816–36. 10.1007/s10995-012-1058-z 22714797

[22] Eslier M Deneux-Tharaux C Sauvegrain P Schmitz T Luton D Mandelbrot L. Association between migrant women's legal status and prenatal care utilization in the PreCARE cohort. Int J Environ Res Public Health. (2020) 17:7174. 10.3390/ijerph17197174 33007972 PMC7579291

[23] Partridge S Balayla J Holcroft CA Abenhaim HA. Inadequate prenatal care utilization and risks of infant mortality and poor birth outcome: a retrospective analysis of 28,729,765 US deliveries over 8 years. Am J Perinatol. (2012) 29:787–93. 10.1055/s-0032-1316439 22836820

[24] Gomes do Espirito Santo ME Perrine AL Bonaldi C Guseva-Canu I. Caractéristiques et état de santé des femmes sans-domicile nées en France et à l'étranger: résultats de l'enquête Insee-Ined 2012. Rev DÉpidémiol Santé Publique. (2018) 66:135–44. 10.1016/j.respe.2017.11.005 29429602

[25] Radimer KL. Measurement of household food security in the USA and other industrialized countries. Public Health Nutr. (2002) 5:859–64. 10.1079/PHN2002385 12633509

[26] McDowell I. Measuring Health: A Guide to Rating Scales and Questionnaires. Oxford: Oxford University Press (2006). p. 765.

[27] Chauvin P Parizot I. Les inégalités sociales et territoriales de santé dans l'agglomération parisienne. Paris: Une analyse de la cohorte Sirs (2005). p. 106.

[28] Patten SB. Performance of the Composite International Diagnostic Interview Short Form for major depression in community and clinical samples. Chronic Dis Can. (1997) 18:109–12. 9375257

[29] Sheehan DV Lecrubier Y Sheehan KH Amorim P Janavs J Weiller E. The Mini-International Neuropsychiatric Interview (M.I.N.I.): the development and validation of a structured diagnostic psychiatric interview for DSM-IV and ICD-10. J Clin Psychiatry. (1998) 59 Suppl 20:22–33; quiz 34–57. 9881538

[30] Blondel B Lelong N Kermarrec M Goffinet F National National Coordination Group of the National Perinatal Surveys. Trends in perinatal health in France from 1995 to 2010. Results from the French National Perinatal Surveys. J Gynecol Obstet Biol Reprod. (2012) 41:e1–15. 10.1016/j.jgyn.2012.04.014 22613118

[31] Bickel G Nord M Price C. Guide to Measuring Household Food Security. Washington, DC: US Department of Agriculture (2000). p. 82.

[32] Haute Autorité de Santé (HAS). Suivi et orientation des femmes enceintes en fonction des situations à risque identifiées. Paris: HAS (2016). p. 42. 10.1016/j.gyobfe.2007.11.010 18323004

[33] Beran TN Violato C. Structural equation modeling in medical research: a primer. BMC Res Notes. (2010) 3:267. 10.1186/1756-0500-3-267 20969789 PMC2987867

[34] Andersen RM. Revisiting the behavioral model and access to medical care: does it matter? J Health Soc Behav. (1995) 36:1–10. 10.2307/2137284 7738325

[35] Vandentorren S Vuillermoz C. Unmet healthcare needs in homeless women with children in the Greater Paris area in France. PLoS ONE. (2017) 8:184138. 10.1371/journal.pone.0184138 28877209 PMC5587267

[36] Campbell DJT O'Neill BG Gibson K Thurston WE. Primary healthcare needs and barriers to care among Calgary's homeless populations. BMC Fam Pract. (2015) 16:139. 10.1186/s12875-015-0361-3 26463577 PMC4603688

[37] Baer RJ Altman MR Oltman SP Ryckman KK Chambers CD Rand L. Maternal factors influencing late entry into prenatal care: a stratified analysis by race or ethnicity and insurance status. J Matern Fetal Neonatal Med. (2019) 32:3336–42. 10.1080/14767058.2018.1463366 29631462

[38] Vanden Broeck J Feijen-de Jong E Klomp T Putman K Beeckman K. Antenatal care use in urban areas in two European countries: Predisposing, enabling and pregnancy-related determinants in Belgium and the Netherlands. BMC Health Serv Res. (2016) 16:337. 10.1186/s12913-016-1478-3 27485241 PMC4970209

[39] Gelberg L Gallagher TC Andersen RM Koegel P. Competing priorities as a barrier to medical care among homeless adults in Los Angeles. Am J Public Health. (1997) 87:217–20. 10.2105/AJPH.87.2.217 9103100 PMC1380797

[40] Gordon AC Lehane D Burr J Mitchell C. Influence of past trauma and health interactions on homeless women's views of perinatal care: a qualitative study. Br J Gen Pract J R Coll Gen Pract. (2019) 69:e760–7. 10.3399/bjgp19X705557 31501164 PMC6733590

[41] Hunt SM McKenna SP McEwen J Backett EM Williams J Papp E. Quantitative approach to perceived health status: a validation study. J Epidemiol Community Health. (1980) 34:281–6. 10.1136/jech.34.4.281 7241028 PMC1052092

[42] Kline RB. Principles and Practice of Structural Equation Modeling, Fourth Edition. New York, NY: Guilford Publications (2015). p. 553.

[43] Muthén B. A general structural equation model with dichotomous, ordered categorical, and continuous latent variable indicators. Psychometrika. (1984) 49:115–32. 10.1007/BF0229421032833145

[44] Beauducel A Herzberg P. On the performance of maximum likelihood versus means and variance adjusted weighted least squares estimation in CFA. Struct Equ Model Multidiscip J. (2006) 13:186–203. 10.1207/s15328007sem1302_2

[45] Carayol M Bucourt M Cuesta J Blondel B Zeitlin J. Les femmes de Seine-Saint-Denis ont-elles un suivi prénatal différent de celui des autres femmes d'Île-de-France? J Gynécologie Obstétrique Biol Reprod. (2015) 44:258–68. 10.1016/j.jgyn.2014.02.006 24702967

[46] Blondel B Gonzales L. Raynaud P. Enquête nationale périnatale, rapport. Paris: INSERM (Institut National de la Santé et de la Recherche Médicale) (2016). p. 317.

[47] Rico Berrocal R. En quête de soins en contexte de précarité: suivis ethnographiques de femmes enceintes en Île-de-France. These (2021). p. 254.

[48] Allen D Feinberg E Mitchell H. Bringing life course home: a pilot to reduce pregnancy risk through housing access and family support. Matern Child Health J. (2014) 18:405–12. 10.1007/s10995-013-1327-5 23820672

[49] Stein JA Andersen R Gelberg L. Applying the Gelberg-Andersen behavioral model for vulnerable populations to health services utilization in homeless women. J Health Psychol. (2007) 12:791–804. 10.1177/1359105307080612 17855463

[50] Interlogement 93. Le 115 pour berceau. Livre Blanc. (2012). p. 16.

