# Supplementary material for: Social determinants of inadequate prenatal care utilization in sheltered homeless mothers in the Greater Paris area in France
Source: Front Public Health. 2023 Mar 21;11:1080594. doi: 10.3389/fpubh.2023.1080594 (PMC10071020; doi:10.3389/fpubh.2023.1080594)
Supplement: Supplementary file 1 [file Table_1.DOCX]

Supplementary material

S1 Table : Characteristics of homeless mothers with at least one child under one year old (N=121) with and without missing data, ENFAMS Cross-sectional survey, Greater Paris area, 2013.

| **Characteristics** | Women with missing data (N=121) | | Women with complete data (N=95) | | |
| --- | --- | --- | --- | --- | --- |
|  | %* | 95%CI** | | %* | 95%CI** |
| Age at the beginning of pregnancy (in years)¹ | 29.7 | (27.5 ; 31.9) | | 29.9 | (27.4 ; 32.5) |
| Education level:   - Never attended school - Primary - Secondary - Higher | 12,0  8.7  56.6  22.7 | (3.9 ; 20.0)  (3.2 ; 14.2)  (38.7 ; 74.2)  (3.9 ; 41.4) | | 10.8  6.9  59.4  22.4 | (3.4 ; 18.2)  (1.4 ; 12.5)  (39.5 ; 79.6)  (1.3 ; 44.6) |
| Difficulty understanding, reading and speaking French language | 55.7 | (41.0 ; 69.7) | | 54.9 | (37.2 ; 72.6) |
| Professional status :   - Employed - Unemployed - Inactive | 13.4  30.8  55.8 | (0.0 ; 28.3)  (18.8 ; 42.8)  (40.0 ; 71.6) | | 15.2  31.5  53.3 | 1. ; 33.1)   (17.4 ; 45.6)  (34.7 ; 71.8) |
| Monthly income per consumption unit (in euros) ¹ : | 282.4 | (189.7 ; 375.1) | | 303.3 | (195.8 ; 410.9) |
| Income : Absence | 30.0 | (17.0 ; 43.0) | | 12.1 | (12.1 ; 41.1) |
| Below poverty line | 98.8 | (97.4 ; 100,0) | | 99.3 | (97.7 ; 100.0) |
| Health insurance :   - No - AME² - CMU² - General social security | 10.0  33.4  48.2  8.3 | (3.6 ; 16.4)  (19.6 ; 47.3)  (30.5 ; 65.9)  (2.6 ; 14.1) | | 9.2  29.8  52.8  8.1 | - 1. ; 15.6)   (15.5 ; 44.2)  (33.3 ; 72.4)  (1.5 ; 14.7) |
| Time of first episode of homelessness:   - More than a year before pregnancy - Less than one year before pregnancy - In the 1^st^ trimester of pregnancy - In the 2^nd^ trimester of pregnancy - In the 3^rd^ trimester of pregnancy - After pregnancy | 34.0  16.9  14.8  8.5  15.4  10.4 | (15.4 ; 52.6)  (8.3 ; 25.4)  (4.6 ; 24.9)  (3.2 ; 13.9)  (7.2 ; 23.6)  (4.2 ; 16.6) | | 35.9  13.2  15.4  7.9  16.0  11.6 | (13.9 ; 57.8)  (5.4 ; 21.1)  (3.3 ; 27.5)  (1.8 ; 14.0)  (5.9 ; 26.0)  (4.5 ; 18.7) |
| Housing instability in the 2^nd^ and 3^rd^ trimesters of pregnancy*** | 71.0 | (54.5 ; 88,6) | | 74.5 | (54.1 ; 94.1) |
| Mother’s birthplace:   - France - Outside of France | 8.5  91.5 | (3.9 ; 13.1)  (86.9 ; 96.1) | | 7.6  92.4 | - 1. ; 12.6)   (87.4 ; 97.3) |
| Administrative status:   - Not regularized - Regularized - French citizen | 35.3  53.2  11.5 | (21.7 ; 48.9)  (38.0 ; 68.4)  (6.1 ; 16.8) | | 29.7  60.2  10.1 | (15.2 ; 44.2)  (43.6 ; 76.8)  (3.8 ; 16.4) |
| Length of immigration before the beginning of pregnancy   - < 6 months - > 6 months - French citizen | 8.5  68.8  22.7 | (3.9 ; 13.5)  (56.7 ; 80.8)  (12.5 ; 32.9) | | 7.6  69.9  22.5 | (2.7 ; 12.6)  (56.2 ; 85.7)  (10.7 ; 34.2) |
| Two or more children under three years old living with mother: | 26.4 | (13.8 ; 39.1) | | 27.7 | (13.3 ; 42.1) |
| Food insecurity :   - Low - Moderate or severe | 51.8  48.2 | ( 34.5 ; 69.1)  (30.9 ; 65.5) | | 55.6  44.4 | (37.1 ; 74.1)  (25.9 ; 62.9) |
| Single Mother | 33.4 | (19.8 ; 47.1) | | 28.9 | (14.1 ; 43.6) |
| PTSD in the previous 12 months | 18.8 | (7.4 ; 30.2) | | 16.1 | (3.4 ; 28.8) |
| Sexually abused | 14.3 | (4.6 ; 24.0) | | 10.4 | (0.4 ; 20.5) |
| Refused AME² or CMU² | 25.3 | (13.2 ; 37.4) | | 23.8 | (10.1 ; 37.4) |
| Unsatisfactory health practitioner engagement | 11.7 | (4.2 ; 19.2) | | 10.3 | (1.6 ; 18.9) |
| Self-perceived general health status:   - Poor/ Very poor - Moderate - Very good/ good | 6.6  37.0  56.4 | (0.0 ; 15.4)  (19.8 ; 54.1)  ( 41.5 ; 68.2) | | 3.8  37.3  58.9 | 1. ; 8.8)   (16.8 ; 57.2)  (23.6 ; 74.3) |
| Primiparous | 31.0 | (19.5 ; 42.4) | | 30.1 | (17.1 ; 43.1) |
| No gynaecological visit | 27.6 | (15.0 ; 40.3) | | 25.2 | (11.8 ; 38.6) |
| Unmet healthcare needs | 20.8 | (10.9 ; 30.8) | | 22.6 | (10.1 ; 35.0) |
| Difficulty of transportation | 43.3 | (10.9 ; 60.8) | | 41.8 | (23.9 ; 59.6) |

* weighted values

**95% confidence interval

¹ Mean

*** Moved home at least once during pregnancy

²AME: free state-based medical aids, CMU: free complementary health insurance for low-income earners

S2 Table : Weighted correlations between indicators of each latent variable of the theoretical measurement model of inadequate PCU in homeless mothers (N=95), cross-sectional ENFAMS survey, Greater Paris area, 2013.

| **Education** | 1 | 2 |  |
| --- | --- | --- | --- |
| 1. Difficulty understanding, reading and speaking French language | 1.00 | 0.23 |  |
| 2. level of education | 0.23 |  |  |
| **Migration trajectory** | 3 | 4 |  |
| 3. African origin | 1.00 | 0.30 |  |
| 4. Immigrant in France for less than six months | 0.30 |  |  |
| **Competitiveness between needs** | 5 | 6 | 7 |
| 5. Food insecurity | 1.00 |  |  |
| 6. Single mother | 0.36 | 1.00 |  |
| 7. More than one dependent child under three years old | 0.18 | 0.07 | 1.00 |
| **Victimization** | 8 | 9 |  |
| 8. Suffered sexual violence | 1.00 |  |  |
| 9. Post-traumatic stress disorder in the 12 months before the study interview | 0.52 | 1.00 |  |
| **Discrimination** | 10 | 11 |  |
| 10. Unsatisfactory care by a health practitioner | 1.00 |  |  |
| 11. Refused for CMU or AME | 0.05 | 1.00 |  |
| **Experience with the healthcare system** | 12 | 13 | 14 |
| 12. Unmet healthcare needs | 1.00 |  |  |
| 13. Never consulted a gynaecologist | 0.01 | 1.00 |  |
| 14. Nulliparous | 0.15 | 0.16 | 1.00 |
| **Self-perceived health status** | 15 | 16 | 17 |
| 15. Psychological self-perceived health status | 1.00 |  |  |
| 16. Physical self-perceived health status | 0.43 | 1.00 |  |
| 17. General self-perceived health status | 0.37 | 0.28 | 1.00 |


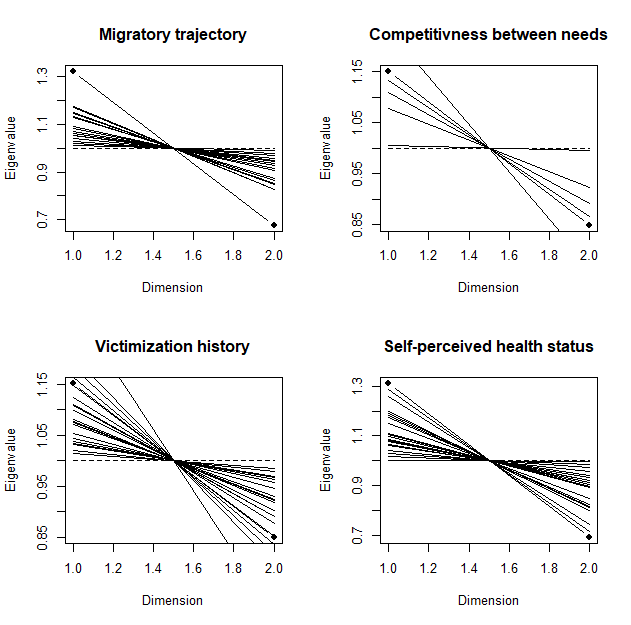


S1 Figure : Scree plot set of latent variables retained in the measurement model of inadequate PCU in homeless pregnant mothers (N=95), cross-sectional ENFAMS survey, Greater Paris area, 2013.
